# A Flow-Measuring Algorithm of Arc-Bottomed Open Channels through Multiple Characteristic Sensing Points of the Flow-Velocity Sensor in Agricultural Irrigation Areas

**DOI:** 10.3390/s20164504

**Published:** 2020-08-12

**Authors:** Yu Han, Tongshu Li, Shiyu Wang, Jian Chen

**Affiliations:** 1 College of Water Resources & Civil Engineering, China Agricultural University, 17 Tsinghua East Rd., Haidian District, Beijing 100083, China; yhan@cau.edu.cn (Y.H.); cau_litongshu@163.com (T.L.); Shiyu_Wang07@163.com (S.W.); 2 College of Engineering, China Agricultural University, 17 Tsinghua East Rd., Haidian District, Beijing 100083, China

**Keywords:** open channel, flow measuring algorithm, discharge, characteristic sensing point

## Abstract

Precise flow measurement in the open channel is a key prerequisite to implementation of modern agricultural efficient water use. The channel with an arc-bottomed shape is the most common channel type in irrigation area at present. The paper has verified the log-law is along the normal line rather than along the vertical line in arc-bottom channel. By conducting the velocity distribution log-law, this paper derives the expression of the multiple characteristic sensing points location of the flow-velocity sensor in the channel section, which is along the normal line. Based on this, a new algorithm to estimate the discharge of the arc-bottomed channel flow is proposed. We have also developed the experiment of the arc-bottomed channels (including semicircular channels, arc-bottom trapezoidal channels and U-shaped channels) and utilize the data to verify the method. The results indicate that the sensing locations expression of the flow velocity measuring sensor such as acoustic doppler velocimetry and propeller is suitable for improving discharge estimation’s accuracy of the arc-bottomed channels. This method could be extensively used in estimating discharge of irrigation and drainage channels in agricultural water conservancy projects. It will enhance the efficiency and accuracy of water resources management departments in irrigation areas, which also meet the strategic requirements of agricultural sustainable development.

## 1. Introduction

Estimating the discharge of open channels is an essential problem in water-resources engineering [1]. As important irrigation and drainage channels, the arc-bottomed channel has the characteristics of large flow capacity, strong sediment transport capacity and significant seepage control effect [2]. Moreover, due to its opening width is narrower than trapezoidal cross-section, a certain extent cultivated land area can be saved. The arc-bottomed channels, such as semicircular, trapezoidal with arc-bottom and U-shaped channel cross-section, have been widely used in irrigation areas currently. For example, the open channels with an arc-bottomed shape accounts for the majority in Hetao irrigation area in Inner Mongolia of China. However, the flow measurement of the arc-bottomed channel is still in the development stage, and the measuring technology has not yet been solved.

As is known, velocity distribution is critical to the calculating the discharge in channels [3]. Since Prandtl put forward the concept of the boundary layer in 1904, Keulegen first proposed that the log-law could describe the velocity distribution of fully developed uniform turbulence in an open channel in 1938 [4,5]:

(1) $$\frac{u}{u_{*}}=\frac{1}{\kappa }\ln\frac{u_{*}\cdot y}{\nu }+B$$

where *u* is velocity, κ is the von Kármán constant, ν is the coefficient of kinematic viscosity and *B* is constant. Here, $u_{*}$ is friction velocity, which reflects the shear stress of the open channel. It is calculated according to the resistance balance of uniform open channel flow.

Considering the influence of the side wall, the formula of friction velocity [6] is,

(2) $$u_{*}=\sqrt{gRS}$$

where *g* is gravitational acceleration, *R* is hydraulic radius and *S* is hydraulic gradient.

Nikuradse carried out an artificial sand rough pipe flow experiment by pasting sand on the wall of a pipe [7]. His result gave that the values of parameters κ and *B* in the log-law are 0.4 and 5.5. After that, other experimenters also gave different parameter values by doing experiments [8,9,10]. In this paper, we will make mathematical derivation and further analysis based on the parameter values in log-law given by Nikuradse, that is,

(3) $$\frac{u}{u_{*}}=2.5\ln\frac{u_{*}\cdot y}{\nu }+5.5$$

Numerous researchers have attempted to give methods for estimating discharge of arc-bottomed open channels in recent years. Some of them have proposed the flow calculation formula to measure the discharge. Dey [11] presented a model that could describe a free overfall from smooth circular channels with flat base and he computed the upstream discharge through the streamline curvature at free surface with the model. Yang et al. [12] established exponent formula for horizontal velocity distribution properties and two-power law for vertical velocity distribution properties. They also put forward the midline three-point for flow measurement of U-shaped channel. Hu et al. [13] used the volume-of-fluid model to show that the parabolic flume is a structure that effectively measures the discharge of U-shaped channels. Moreover, they derived a formula for calculating discharge that is applicable to U-shaped channels of various slopes. Besides these, there are a few researchers who designs flumes, weirs and gates for flow measurement. Samani [14] designed three simple flumes for flow measurements in different cross-section open channels. He combined the Pi Theorem principle with laboratory scale physical models and developed the calibrated equations. Mohammadzadeh-Habili et al. [15] investigated a new weir entitled of quarter-circular crested weir for flow measurement. They determined that the discharge coefficient of the weir was constant through conducting experiments. Vatankhah et al. [16] proposed solid semicircular flap gates which is used to measure discharge along the circular channels under free flow condition. However, its leakage is inevitable and cannot be quantified.

Both the formula method and the weir-gate method exist problems in application scope and simplicity. Thus, this study wants to give a reliable and simple method in order to provide a good idea of flow measurement. Unlike previous studies, this paper proposes a new algorithm to estimate the discharge through multiple characteristic sensing points of the flow-velocity sensor in open channels with an arc-bottom by using velocity distribution log-law. First of all, the log-law is verified along the normal line rather than along the vertical line through comparison. Then, the paper gives theoretical expression of multiple characteristic sensing points location of the flow-velocity sensor and the formula for calculating channel discharge. Finally, based on the derived results, the discharge can be quickly obtained by using the sensor to measure the velocity of the characteristic points and substituting the discharge calculation formula. The relevant experimental design is carried out, involving three cross-section types of channels: semicircle, trapezoid with an arc-bottom and U-shaped. Through comparative analysis, it is considered that the multiple characteristic sensing points of the flow-velocity sensor can be applied to the flow measurement of arc-bottomed channels.

## 2. Methodology

**Figure 1:**
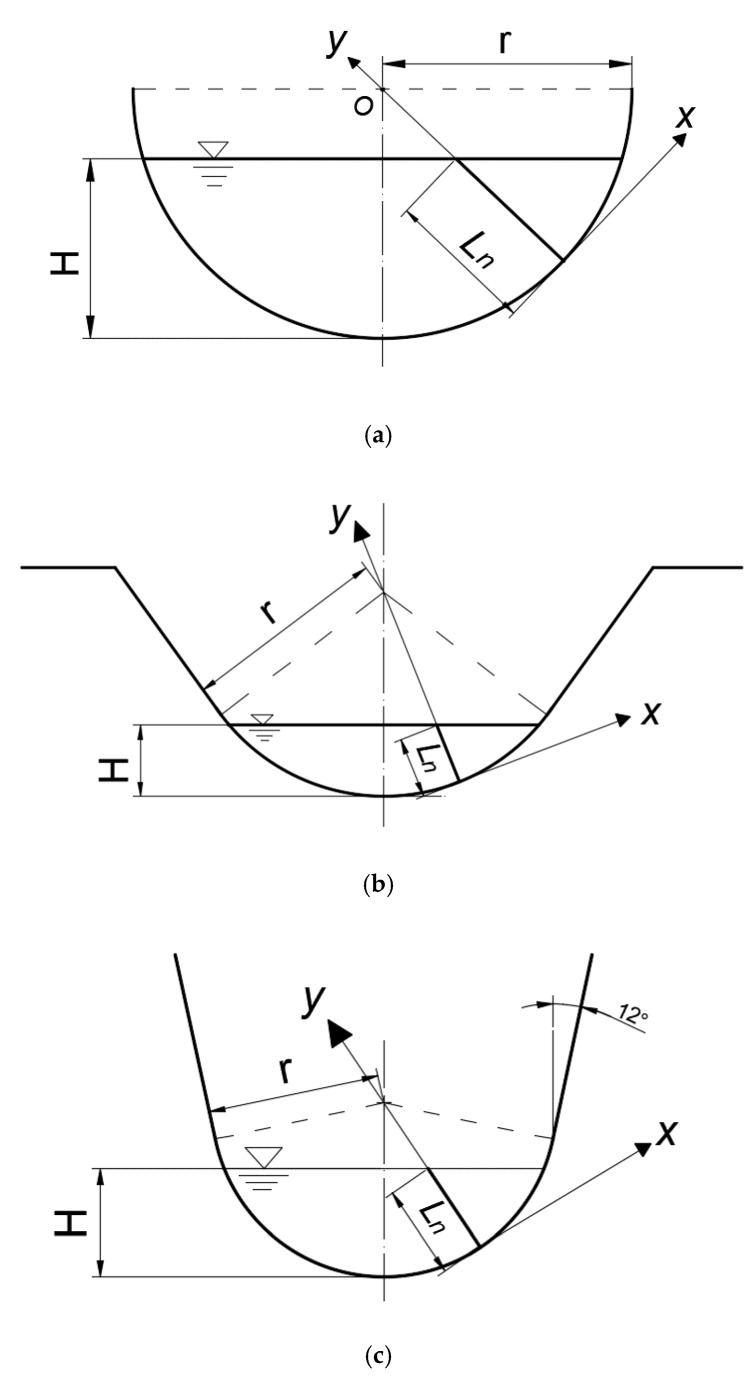
Three common kinds of cross-section types of channels with an arc-bottom. (**a**) Semicircle; (**b**) arc-bottom trapezium; (**c**) u-shaped. Here, *x* and *y* are the axes of the coordinate system, *H* is water depth, *r* is radius of the arc, and *L_n_* is the length of the underwater part of the line.

The transportation direction of flow surplus energy is along the relative shortest geometric distance to the boundary [17]. For the arc-bottomed channel, due to its channel boundary is curvilinear, we can connect the center of the circle and any point in the channel cross-section and extend it to get the intersection point with the side wall. Then, it can be obtained that the length between these two points is the relative shortest geometric distance of energy dissipation. Hence, the mathematical derivation in methodology section is based on the velocity distribution along the normal line direction. The research object of this paper is arc-bottom channel. As shown in Figure 1, the common types of arc bottom channel are semicircle, arc-bottom trapezium and U-shaped.

### 2.1. The Location of Characteristic Sensing Point of the Flow-Velocity Sensor in Arc-Bottom Channel

In this paper, because of the practical diversity of arc-bottomed channel forms, the derivation is based on the simplest form of the arc-bottomed channel. The simplified standard semicircular channel section diagram is analyzed.

**Figure 2:**
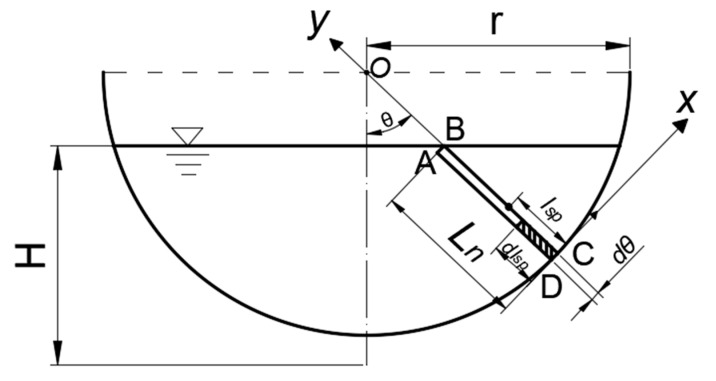
The semicircular channel is taken as an example to analyze and deduce. Here, *θ* is the angle between the center line and the line passing through the circle center O. *l_sp_* is the distance between characteristic sensing point to the sidewall.

In Figure 2, there is a straight line, which is angled with the center line as *θ*. The rectangle ABCD is *L_n_* in length and *dθ* in width. Since *dθ* is infinitely close to zero, the discharge through the rectangle ABCD can be expressed as:

(4) $$q=u\cdot A=u\cdot L_{n}\cdot d\theta .$$

Then extracting the shadow rectangle with edge lengths being *dl_sp_* and *dθ*, the discharge of the shadow rectangle is:

(5) $$dq=u\cdot dA=u\cdot d\theta \cdot dl_{sp}.$$

For Equation (5), we integrate *dl_sp_* in the direction of *y*, that is,

(6) $$q^{’}=d\theta \cdot \int_{0}^{L_{n}}udl_{sp}.$$

According to Equation (3), velocity can be shown as:

(7) $$u=u_{*}\cdot (2.5\ln\frac{u_{*}\cdot l_{sp}}{\nu }+5.5),$$

where the meaning of *l_sp_* and y in Equation (3) are the same.

Substituting Equation (7) to Equation (6), it can get:

(8) $$q^{’}=d\theta \cdot \int_{0}^{L_{n}}u_{*}\cdot \left(2.5\ln\frac{u_{*}l_{sp}}{\nu }+5.5\right)\cdot dl_{sp}=d\theta \cdot u_{*}\cdot L_{n}\cdot \left(2.5\ln\frac{u_{*}\cdot L_{n}}{\nu }+3\right).$$

Substituting Equation (7) to Equation (4), it can get:

(9) $$q=d\theta \cdot u_{*}\cdot L_{n}\cdot \left(2.5\ln\frac{u_{*}\cdot l_{sp}}{\nu }+5.5\right).$$

Since q=q’, the expression of *l_sp_* is obtained by simultaneous combining Equations (8) and (9), that is:

(10) $$l_{sp}=\frac{L_{n}}{e},$$

where *L_n_* is the length of the underwater part of the line, *e* is natural constant and *l_sp_* is the distance between characteristic sensing point to the sidewall along the normal line. The characteristic sensing point’ s velocity can represent the average velocity of the measuring line.

### 2.2. Derivation of Estimating Discharge through Multiple Characteristic Sensing Points of the Flow-Velocity Sensor

**Figure 3:**
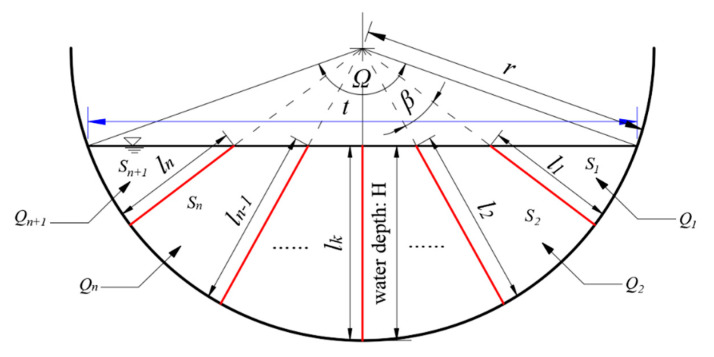
Diagram of estimating discharge with *n*-measuring-lines. Here, *l_1_, l_2_…l_n_* are the lengths of measuring lines. *S*_1_, *S*_2_…*S*_*n*+1_ are sub-areas of cross-section. *Q*_1_, *Q*_2_…*Q*_*n*+1_ are discharges of sub-areas. *Ω* is the total angle. *β* is the angle between each two-measuring line. *t* is the width of water surface.

Since we have obtained the location formulation of characteristic sensing point of the flow-velocity sensor in Section 2.1, we can estimate discharge with multiple characteristic sensing points’ velocities through setting numerous measuring lines. As is shown in Figure 3, there are totally *n* measuring lines equally divided angle *Ω*, the length of which are *l_1_, l_2_…l_n_*. The angle between each two lines is *β*. It can be shown that

(11) $$\Omega =2\arcsin\left(\frac{t}{2r}\right)$$

(12) $$\beta =\frac{\Omega }{n+1},$$

where *t* is the width of water surface, *n* is the number of measuring lines.

The hydraulic radius *R* is expressed as:

(13) $$R=\frac{\Omega r-r\sin\Omega }{2\Omega }.$$

The length of the *k*–th line and the area of the *k*–th area are, respectively,

(14) $$l_{k}=r-r\cdot \frac{\cos\left(\frac{\Omega }{2}\right)}{\cos\left(\frac{\Omega }{2}-k\beta \right)}$$

(15) $$S_{k}=\frac{r^{2}}{2}\cdot \left\{\beta -\frac{\cos^{2}\frac{\Omega }{2}\cdot \sin\beta }{\cos\left(\frac{\Omega }{2}-k\beta \right)\cdot \cos\left[\frac{\Omega }{2}-\left(k-1\right)\beta \right]}\right\}.$$

The discharge of each region can be shown as:

(16) $$Q_{k}=\frac{\delta_{k}+\delta_{k-1}}{2}\cdot S_{k}(2\leq k\leq n),$$

where $\delta_{1}$ is the velocity of characteristic sensing point on the measuring line *l_k_*.

Because *S*_1_ and *S*_*n*+1_ are at the edge of channel, the discharge can be estimated separately,

(17) $$Q_{n+1}=\alpha \cdot \delta_{n}\cdot S_{n+1},$$

where *α* is velocity coefficient along the bank, $\delta_{1}$ is the velocity of characteristic sensing point on the measuring line *l*_1_.

Hence, the discharge *Q* of channel can be calculated through Equation (15),

(18) $$Q=\sum_{k=1}^{n}Q_{k}+Q_{n+1}.$$

## 3. Experimental Setup

**Figure 4:**
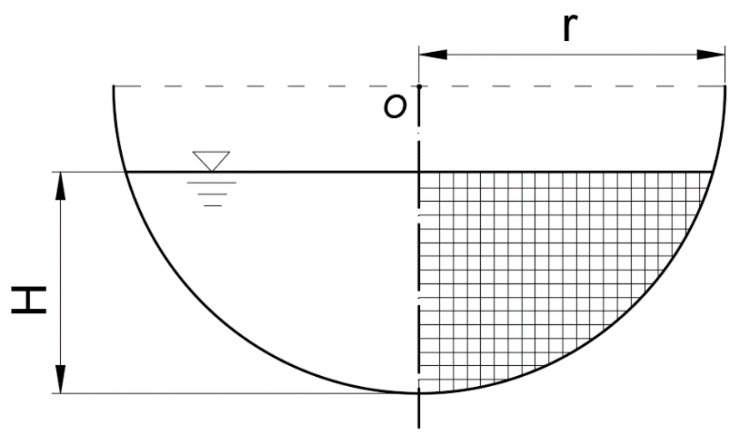
Schematic diagram of measurement points in the experiment (take semicircular channel as an example).

Table 1 has summarized the experimental conditions and relevant parameters of channel. There are three types of channel section in table: semicircular, arc-bottom trapezoidal and U-shaped. The channel cross-sectional shape is shown in Figure 1. In order to increase the reliability, the tests are carried out in different places, such as the Hydraulic Experiment Hall of China Agricultural University (CAU) (Beijing, China), the College of Civil Engineering at the University of Birmingham (UoB) (Birmingham, UK), and the Applied Hydraulics Laboratory at the National Technical University of Athens (NTUA) (Athens, Greece). When the channel bottom slope is certain and the flow is uniform, one discharge *Q* corresponds to one water depth H. Thus, different groups set up in Table 1 are to ensure that the experiment is carried out under the condition of uniform flow. The experiments conducted in CAU uses the flow velocity measuring sensor such as acoustic doppler velocimetry and propeller. Besides that, the experimental instruments also consist of electromagnetic flowmeter, water pump, water stabilizing equipment, frequency converter, etc. We use these measuring sensors to collect three-dimensional velocity data in the channel from bottom upward along the vertical direction to the water surface. As is shown in Figure 4, all the corners of the lattice are points need to be measured.

## 4. Results and Discussion

### 4.1. Analysis along Normal Line Direction

According to Yang and Lim [17], they put forward a concept of energy transportation. They proposed that the direction of energy transportation is along the shortest geometric distance, between the source concerned and the boundary.

**Figure 5:**
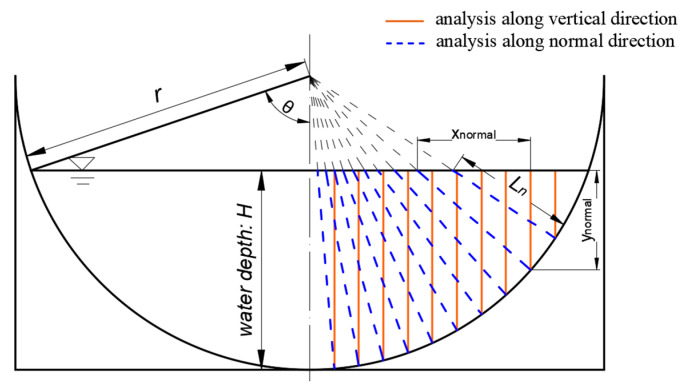
Analysis along vertical or normal direction (red solid line refers to the vertical direction and blue dotted line refers to the normal direction). Here, *x_normal_* and *y_normal_* are the projection lengths of the line in the horizontal and vertical directions.

We used Equation (3) to make a comparative analysis along the vertical direction and the normal direction, respectively under C1 experimental condition. The analysis diagram is shown in Figure 5.

Giving a definition formula of normal slope $k_{normal}$, that is,

(19) $$k_{normal}=\left|\frac{y_{normal}}{x_{normal}}\right|,$$

where $y_{normal}$ and $x_{normal}$ can be seen in Figure 5.

The calculation formula of the average error value in Table 2 is given by,

(20) $$E=\frac{E_{e}-E_{t}}{E_{e}}\times 100\%,$$

where *E* is the average error value, $E_{e}$ denotes the experimental value and $E_{t}$ denotes the theoretical value with the log-law.

Using Equations (19) and (20), we can verify the application conditions of the log-law Equation (3) from two directions, as is shown in Table 2.

It can be seen from Table 2 that when the measuring velocity and the log-law are analyzed for consistency, the error value obtained along the normal direction is much lower than the error value obtained along the vertical direction. This can prove the correctness of the analysis along the normal line in the arc-bottomed channel.

### 4.2. Multiple Characteristic Sensing Points of the Flow-Velocity Sensor in Arc-Bottom Channel

**Figure 6:**
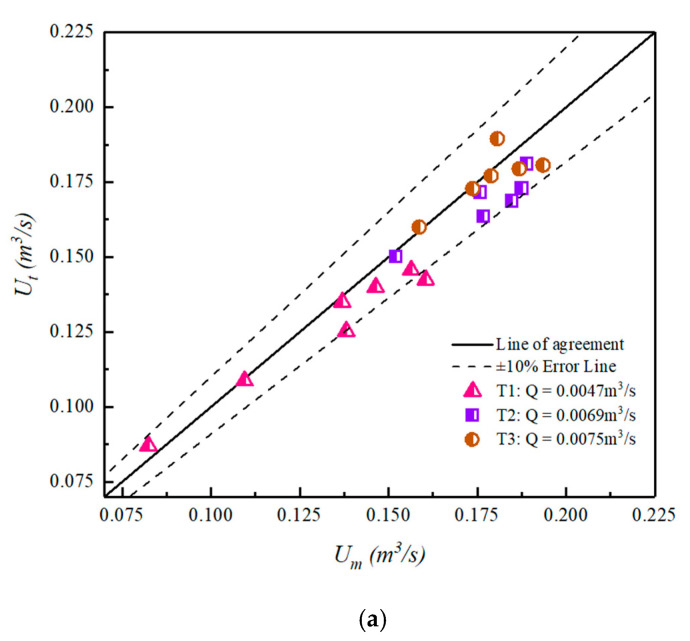

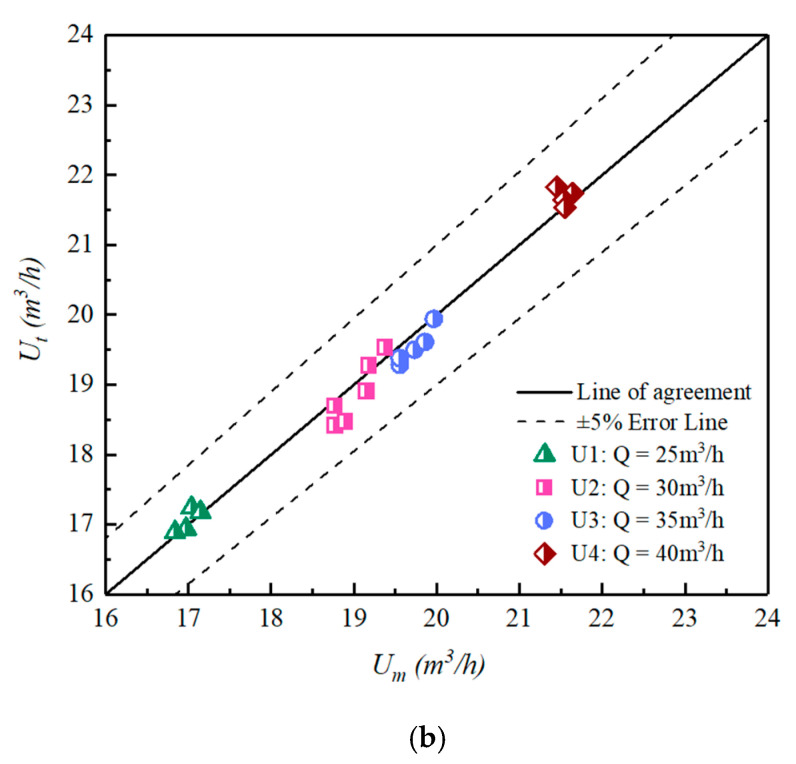
Comparison of measured and theoretical mean velocities: (**a**) T1–T3; (**b**) U1–U4.

Figure 6 is the comparison between the measured and theoretical average velocity values under different experimental conditions along normal direction. The *U_m_* is the average velocity of each measuring point on the normal line, while *U_t_* is obtained by theoretical calculation. Specifically, the location of characteristic sensing point is calculated by formula first and then the *U_t_* is obtained by substituting the location to log-law.

From the comparison chart of mean radial velocity, we can get that the measured value of flow velocity is well-coincident with the theoretical value. Despite some fluctuations in individual test points, most of them are within the 10% error. The statistical rules of these data points can still prove the accuracy of the theoretical formula in Section 2.1. Thus, the velocity of characteristic sensing point is considered to represent the average velocity of normal direction.

### 4.3. Estimating Discharge through Multiple Characteristic Sensing Points of the Flow-Velocity Sensor

**Figure 7:**
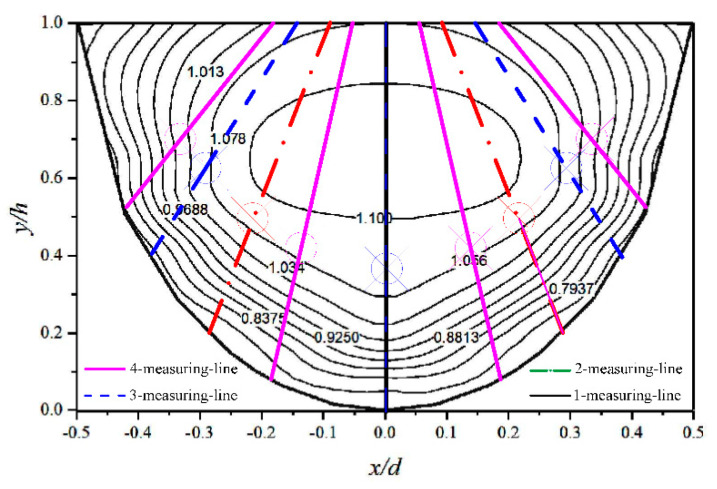
Schematic diagram of flow measurement with different number of measuring lines (C2).

As can be seen from the above, the calculation formula of discharge has been given in Section 2.2. As is shown in Figure 7, for C2 experimental condition, we set four combinations of measuring lines.

When only one measuring line is used to calculate the cross-section discharge, the measurement result is smaller than the displayed result on the flowmeter, which the relative error is 21.61%. Then, when two measuring lines are set on the cross-section, the relative error is reduced to 9.91%. The relative error is 4.18% when the number of measuring lines is increased to three and 2.95% when the number of measuring lines is four. We can conclude that the number of measuring lines is directly related to the discharge calculation in arc-bottom channel. It can be inferred that with the increase of the number of measuring lines, the relative error between the calculated discharge and actual discharge will be decreasing.

**Figure 8:**
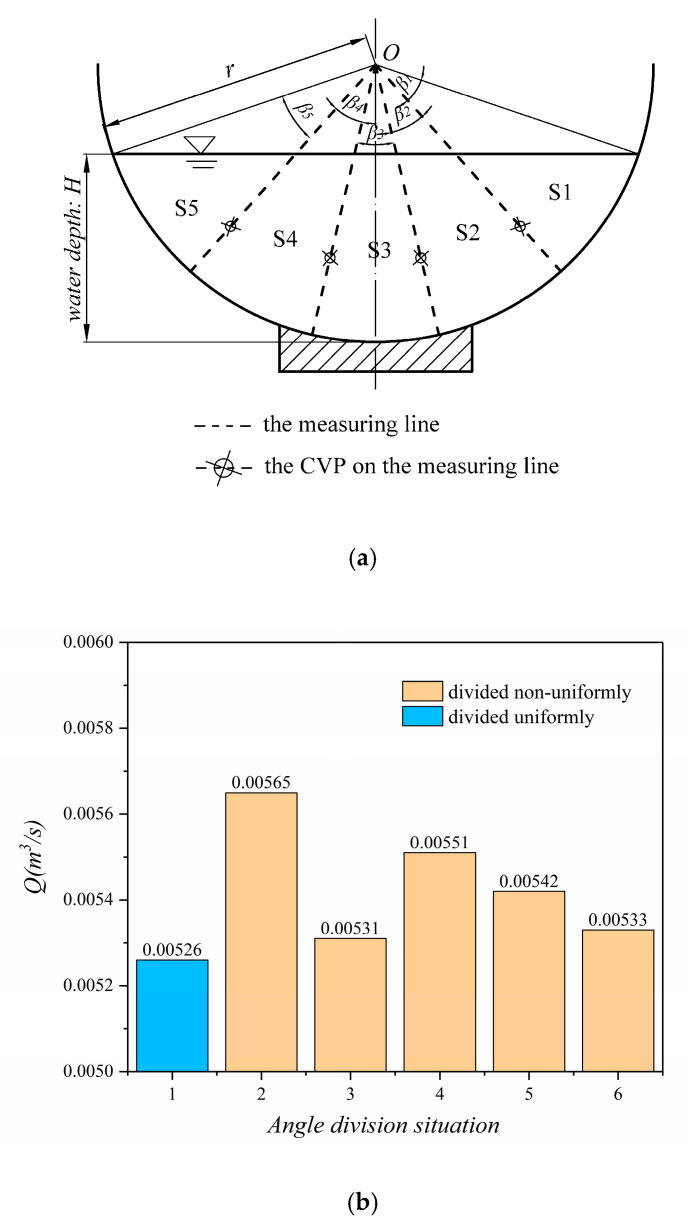
Estimating discharge of different angle combinations of four measuring lines (C1): (**a**) The angle *Ω* is divided to different sub-angles with four measuring lines. (**b**) The relationship between angle division situation and calculated discharge.

Then, the analysis is based on fixing the number of measuring lines and change the angle *β* between them. We take the C1 condition as the example. As is shown in Figure 8a, there are four measuring lines and the angles are randomly divided. Since *Q* = 0.005 m^3^/s in C1, Figure 8b shows that only the angle is divided uniformly, the calculated discharge is closest to the actual discharge.

Therefore, considering the relative error and operability and simplicity in practice, we conclude that a 4-line method is most suitable for measuring discharge in arc-bottom open channel.

## 5. Conclusions

This study has proposed a new algorithm of estimating discharge of channels through sensors in irrigation area. The principal achievements are summarized below.

(1) We compared the log-law along the normal direction and along the vertical direction. The results show the accuracy with the normal direction. We thought that the analysis should be carried out along the normal direction in the arc-bottomed channel rather than vertical direction,
(2) Based on the log-law, the theoretical expression for calculating the locations of multiple characteristic sensing points in arc-bottom open channel is derived through the formula transformation. Then, we give the mathematical formula to calculate the discharge of the arc-bottomed channels. We can use sensors to measure the velocity of these characteristic sensing points and obtain the discharge. The correctness of the formula for multiple characteristic sensing points in arc-bottom channels is proved by numerous experimental data;
(3) To prove the validity of the flow calculation formula, we estimate the discharge by setting different measuring lines. Compared with the measuring discharge, the calculating discharge can match well. Moreover, the number of measuring lines is positively correlated with the accuracy of flow measurement. When the angles between lines are the same, the number of measuring lines is positively correlated with the accuracy of flow measurement. When the number of measuring lines is fixed, the accuracy of angle uniformity is higher than that of nonuniformity.

Estimating channel discharge can better control the water consumption of irrigation area and regulate the water use of canal system. The multiple characteristic sensing points of the flow-velocity sensor and flow formula proposed by this study provide a theoretical basis for simplifying and quickly estimating the discharge of the arc-bottomed channel. It is an indispensable part of irrigation water management, which has practice-oriented significance in agricultural water management.

## Figures and Tables

**Table 1:** Summary of experimental conditions.

Cross-Sectional Shape	Arc Radius (m)	Bottom Slope	*Q* (m^3^/h)	*H* (m)
Semicircle (C1–C2)	0.120 ^a^	0.001	19	0.0813
	0.240 ^c^		–	0.2152
U-shaped (U1–U4)	0.250 ^b^	0.000667	25	0.123
			30	0.138
			35	0.149
			40	0.162
Arc-bottom trapezium (T1–T3)	0.190 ^b^	0.000667	17	0.0124
			25	0.0149
			27	0.0155

^a^ Experiment data from Donald W. Knight’ s paper [18]. ^b^ Experiment data from China Agricultural University. ^c^ Experiment data from Demetriou and Nanou-Giannarou [19].

**Table 2:** Comparison of the application of the log-law in normal and vertical lines, with condition C1: H = 0.0813.

Average Error Value along Normal Direction		Average Error Value along Vertical Direction	
Normal Slope *k_normal_*	Average Error Value *E* (%)	The Distance from Vertical Line to Central Line *Z*/*R*	Average Error Value *E* (%)
11.96	3.35	0.08	6.63
5.92	3.12	0.17	5.92
3.87	3.28	0.25	5.04
2.83	3.15	0.33	4.17
2.18	1.63	0.42	3.64
1.73	2.24	0.50	4.02
1.39	1.07	0.58	3.92
1.12	1.07	0.67	4.88
0.88	2.49	0.75	8.35
0.66	6.53	0.83	14.42
